# An Imperial Union of Nurses

**Published:** 1918-04-27

**Authors:** 


### NURSING AFFAIRS IN IRELAND.

### An Imperial Union of Nurses.

Public thought in Ireland just now is concentrated on conscription, and every variety of passion and motive animates the populace. Underlying the whole matter is apparent the conviction that England's just resentment of Ireland's aloofness from wholehearted participation in the war will not pass away like a mist. It is desirable, however, to point out, and with emphasis, that there are a great many thousands of people in Ireland whose war zeal and enthusiasm surmount every other passion, and whose sacrifice of blood and kindred and treasure are second to none. The atmosphere affects every department of work, including the nursing world—a fact which the College of Nursing will doubtless recognise. A very substantial contingent, including the best of the younger Irish nurses, are in France and at other battle-fronts. Those who have gone to do their part in the war demonstrate by their action their enlightenment, as well as their physical, moral, and mental robustness. To such as these, primarily, I suggest that the work of propaganda should be entrusted, and that an effort should be made to enlist their services despite the fact that they are busily engaged with pressing duty.

What have we left at Home? There is no disputing the fact that the dominant personalities of the irreconcilable school consist almost, if not altogether, of old women—some of them very old women and some untrained. I make this comment in no unchivalrous spirit. Age is honourable, and, so far as they are trained nurses, their records of training and experience are good. But they are entirely out of touch with the hopes, ambitions, and requirements of the rising generation, and their habit of mind, moreover, is utterly and completely anti-democratic. Their careers as workers are at an end, and their only remaining desire in a world where they have grown obsolete is to preserve at all costs their individual identity and prestige. It is not in accord with the fitness of things to leave in such hands the guiding and forming of the destinies of the young. This principle applies always and generally, but it applies with especial force to-day. During the past three years even the young have lived a whole lifetime. Revolution has followed revolution like the films on a cinema screen. Yesterday the British soldier was the paid servant of the State. To-day he is everyman—cook's son and duke's son—the Empire's manhood. Conscription has lost its stigma: it is national service, the just and reasonable tribute to the Commonwealth. If Ireland stands aloof it may be bad for the Empire, but inevitably it will be worse for Ireland. No man is a gainer by being blind and deaf to duty's call. It is only the young who can properly appreciate the modern trend. A little while ago France, England, and America were on polite terms of acquaintanceship. To-day, not the paid armies of these countries, but the very hearts of them, are pouring out their blood in the common cause of righteousness. The differences of distance and race are being obliterated, and when the battle is over and the victors return to their tents the bond of brotherhood will not be weakened.

In the nursing world the common movement towards union should be quickened, and the most effective stimulant is young blood. The war worker's experience during recent years has added cubits to her stature, and she has proved that her services to the nation are as essential as those of the men-at-arms. Every war nurse, at Home or at the Front, possesses the potentiality of a Florence Nightingale. The mission of the College is to reflect the rational and imperial sentiment of closer brotherhood. If this lofty ideal is to be achieved, the time to work is now while the attention of the whole Empire is riveted on the war. Remember the time-worn sentiment about the tide, and let every war worker be imbued with the importance of taking advantage of the flood.

A Union of Nurses is, in their individual interests, imperatively essential, and for the most practical purposes. It is not a matter of sentiment, but of business. A National and Imperial Union should claim national and imperial recognition, and with this object clearly defined they should labour unceasingly. To my mind, the most conspicuous blunder of the Bedford-Fenwick School is to hold in derision what they are pleased to describe as the lay element. The lay element is nothing more or less than public opinion, without which all effort after progress must of necessity be futile. Suffering from this brain-canker of hostility to public sympathy, the Bedford-Fenwick School have for thirty years kept the profession stagnant. I do not doubt their honest intention, but I am convinced that this obsession is the result of their failure to achieve. A striking proof of this contention is the work done for nursing in India by the Dowager Marchioness of Dufferin, or in Ireland by the Countess of Dudley. The glib and insolent references persistently made by this school to "Society women" demand exposure. It is not merely that they are subtle slanders, but what is of greater importance is that they are calculated irreparably to damage the prospects of hard-working and deserving women, especially as they emanate from avowed champions of democratic rights. If I may offer a personal criticism of the constitution of the College of Nursing, it is that the professional element is too predominant, and the lay element correspondingly insignificant. The function of the laity, or otherwise of the British public, is to bestow on the nursing profession full public recognition of valuable public services in the form of material benefit. The advice to be "independent" savours of the servants' hall, and suggests an impudent response to kindness and good feeling. The important truth for nurses to bear in mind is that they are public servants, discharging duties of immense public mportance. To repel the public who desire to appreciate their services is to ally themselves with flunkeyism in its worst and most offensive form.

The College of Nursing is essentially an institution for the youth, the intelligence, and the ambition of the profession, and the fulfilment of its high mission depends on the ardour of its members. If they are wise in their generation they will court rather than offer affront to the British public.

